# Evaluating investments in electricity resilience: evidence from California’s Public Safety Power Shutoff (PSPS) events

**DOI:** 10.1038/s41598-026-50735-4

**Published:** 2026-09-11

**Authors:** Daniel Thompson, Maham Furqan, Gianluca Pescaroli

**Affiliations:** 1 University College London, London, UK; 2 World Bank, Washington, USA; 3 CCRS, Cambridge Judge Business School, Cambridge University, Cambridge, UK

**Keywords:** Electricity resilience, Electricity investments, Cascading failure and compound events, Public Safety Power Shutoffs (PSPS), Energy and society, Energy science and technology, Engineering

## Abstract

Cascading failures and compound effects in electricity systems are a growing concern for infrastructure resilience, as they increasingly lead to additional hazards (e.g., wildfires), further exacerbating power disruptions. In California, investor-owned utilities (IOUs) have responded to these threats by enacting Public Safety Power Shutoffs (PSPS), which are proactive de-energization of high-risk circuits, to reduce wildfire risk. Although prior research has examined PSPS impacts, little is known about whether physical infrastructure investments have reduced de-energizations on the most frequently affected circuits. This study addresses that gap by evaluating whether circuit-level investments have decreased the frequency or severity of PSPS events in California. We overlay investment and de-energization data at the circuit level to conduct this analysis. Despite limited data for certain investments, early results suggest that these investments are not associated with a reduction in the frequency or severity of de-energizations. Moreover, external wildfire risk factors alone do not seem to explain these patterns, suggesting that internal utility decision-making and other risk factors (e.g., aging infrastructure) play a critical role in which circuits are de-energized. These results underscore the importance of considering alternative investment strategies in physical infrastructure upgrades, like distributed energy resources (DERs), to minimize the impact of de-energizations on the most impacted circuits.

**Supplementary Information:**

The online version contains supplementary material available athttps://doi.org/10.1038/s41598-026-50735-4.

## Introduction

The recombination of technological networks’ vulnerabilities, the accelerating impacts of climate change, and mounting geopolitical instabilities have created an increasing need to enhance existing adaptive capabilities. This may require a shift from scenario-focused remediation to building system-wide resilience, especially when precursors to disruptions are not well understood^1–3^. Enhancing the resilience of critical infrastructure systems plays a central role in this process, since these systems form nodes where common vulnerabilities across socio-technical systems accumulate. When disrupted, they can trigger cascading crises^1,4^ or create new crises, like high-probability, low-impact events^5^.

The cascading effects of crises are especially pronounced in electricity systems, as many other sectors grow increasingly dependent on electricity, while new horizons of risk emerge simultaneously, exacerbating existing crises and creating new ones^6^. California is a prominent example of how electricity resilience is evolving to address emerging threats, where utilities are adapting to the reality that cascading grid crises are not only caused by external hazards like wildfires but can also be triggered by the grid itself, as in the case of wildfire ignitions^7^.

California’s investor-owned utilities (IOUs) have been mandated by state regulators to consider implementing Public Safety Power Shutoffs (PSPS) to address grid-ignited wildfires. PSPS are preemptive circuits or transmission lines de-energizing events intended to reduce grid-induced wildfire ignition risks^8,9^. Although these events may reduce ignition risks, they have introduced a secondary consequence on electricity resilience by causing recurrent electricity interruptions for customers on circuits frequently impacted by de-energizations.

The IOUs have made targeted investments in circuit-level infrastructure upgrades to address the second-order impacts of de-energizations on specific circuits, particularly in areas with frequent PSPS activity, particularly for California’s three largest IOUs, including Pacific Gas and Electric (PG&E), Southern California Edison (SCE), and San Diego Gas and Electric (SDG&E)^8,9^. PG&E’s Enhanced Powerline Safety Settings (EPSS) aimed to reduce wildfire ignition risk as well as grid hardening, vegetation management, and installing safety technologies – all targeted to minimize overall grid vulnerabilities. In a recent news press, the company noted that EPSS reduced average outages by 17%^10^. SCE’s publicly stated goal has been similar – to reduce wildfire ignition risk, with a secondary goal to limit PSPS events^11^. SDG&E similarly noted that their circuit investments (e.g., infrastructure hardening, covered conductors, etc.), can help reduce PSPS event needs^12,13^. In a recent comment to the Office of Energy Infrastructure Safety, SDG&E mentioned that their wildfire mitigation investments, e.g., covered conductors, can help minimize PSPS risk.

This paper traces the effectiveness of the IOUs’ investment in response to these second-order effects of electricity resilience by asking the question: *have circuit-level investments to minimize de-energizations from PSPS events demonstrably reduced the frequency or severity (measured in terms of duration) of circuit-level de-energizations?*

Answering this question will contribute to research on PSPS events and research around physical infrastructure investment aimed at improving the resilience of electricity systems. Research on PSPS remains relatively limited, which may be due to its recent adoption by the IOUs^14,15^. Existing studies have primarily focused on estimating impacts on electricity customers^16,17^, quantifying economic losses^9,18^, or conducting simulations^19^. Some research has examined the regulatory trade-offs associated with PSPS implementation^20^, but few studies have evaluated how utilities have attempted to mitigate the secondary impacts of PSPS de-energizations on electricity customers, particularly at the circuit level. This study also contributes to research that assesses how physical upgrades can enhance the resilience of electricity systems. Where prior work generally has focused on policy-level analysis^21^, general review^22^, or theoretical modeling^23^, this study offers an empirical example of a resilience measure in practice.

The results section is divided into two sections. The first section compares PSPS de-energizations on circuits with and without prior infrastructure upgrades to assess the impact of investment on reducing the frequency or severity of disruptions. The second section uses geospatial analysis to evaluate whether these de-energizations were driven by external wildfire factors only (i.e., not entirely the fault of the investments) or also influenced by internal utility decision-making. This is followed by a discussion and conclusion section that links the findings to existing studies and identifies areas for future research. The methods section at the end details the approach used for both results sections.

## Results

### Comparing circuits’ performance - investment vs. no investment

Results were calculated by comparing the recurrence performance of circuits that experienced at least one de-energization during the baseline period of 2018–2022 with the recurrence performance of the same circuits during the subsequent period of 2023–2024. Circuits were classified according to the investment types received during the baseline period. To account for circuits receiving multiple investment types, the analysis was performed using both mutually exclusive investment classifications as well as independent evaluation of individual investment categories.

Across all utilities, 1,191 circuits were de-energized at least once during 2018–2022, with 168 circuits experiencing at least one repeat de-energization during the subsequent observation period from 2023 to 2024. Circuits that had received no physical investment had a recurrence rate of 11% de-energizations, which constituted 24% reduction in the proportion of circuits de-energized (Table 1). Circuits across all physical investment categories exhibited higher recurrence rates during 2023–2024. Circuits with a combination of investments had a recurrence rate of approximately 26%, or a multiple of 1.8 more than expected. Similarly, circuits with sectionalizing had a recurrence rate of approximately 41%, or a multiple of 2.9 more than expected. Circuits that had received overhead hardening exhibited a recurrence rate of 23%, or a multiple of 1.6 more than expected. Circuits that had been undergrounded had a recurrence rate of 40%, with a multiple of 2.8 more than the expected baseline. Due to the small baseline sample size of 5 total circuits for undergrounding, however, the results from this test should be considered exploratory at best (Table 1).

**Table 1:** Frequency-based analysis, results for all utilities.

Investment category	Baseline (2018–2022) no. of circuits	Baseline share of circuits	Repeat (2018–2022) no. of circuits	Repeat share of circuits observed	Repeat share observed over expected	Recurrence rate	Recurrence performance ratio	Recurrence performance difference	P (Fisher’s exact)
No investment	995	84%	106	63%	0.76	11%	1.00	0.00	2.00E-12
Combination	50	4%	13	8%	1.84	26%	2.44	0.15	0.021
Overhead hardening	61	5%	14	8%	1.63	23%	2.15	0.12	0.056
Sectionalizing	80	7%	33	20%	2.92	41%	3.87	0.31	4.69E-10
Undergrounding	5	0%	2	1%	2.84	40%	3.75	0.29	0.148

When disaggregating results by utility, recurrence performance patterns were directionally consistent but less statistically significant due to smaller cohort sizes and uneven distribution of investment categories. For example, the 70 circuits that had received sectionalization in utility A had a recurrence rate of approximately 41.4% (a multiple of approximately 4 more than utility A’s baseline), compared to a recurrence rate of 29% (24x multiple) across the 4 circuits that had been sectionalized in utility C (Table 2).

**Table 2:** Frequency-based analysis, results for each utility.

Table 3 –Investment category	Utility	Baseline (2018–2022) no. of circuits	Baseline share of circuits	Repeat (2018–2022) no. of circuits	Repeat share of circuits observed	Repeat share observed over expected	Recurrence rate	Recurrence performance ratio	Recurrence performance difference	P (Fisher’s exact)
Combined	A	37	4%	12	10%	2.29	32%	3.09	0.22	0.003
No physical investment	A	733	86%	77	64%	0.74	11%	1.00	0.00	2.56E-11
Overhead hardening	A	15	2%	3	2%	1.41	20%	1.90	0.09	0.457
Sectionalizing	A	70	8%	29	24%	2.93	41%	3.94	0.31	3.29E-09
Undergrounding	A	1	0%	0	0%	0.00	0%	0.00	-0.11	1
Combined	B	12	5%	1	3%	0.54	8%	0.60	-0.05	0.698
No physical investment	B	203	78%	28	70%	0.89	14%	1.00	0.00	0.208
Overhead hardening	B	44	17%	11	28%	1.62	25%	1.81	0.11	0.067
Combined	C	1	1%	0	0%	0.00	0%	0.00	-0.02	1
No physical investment	C	59	78%	1	14%	0.18	2%	1.00	0.00	0.0003
Overhead hardening	C	2	3%	0	0%	0.00	0%	0.00	-0.02	1
Sectionalizing	C	10	13%	4	57%	4.34	40%	23.60	0.38	0.005
Undergrounding	C	4	5%	2	29%	5.43	50%	29.50	0.48	0.040

Despite the overall consistency in the directional pattern of recurrence performance across investment categories across utilities, the large disparities in magnitude suggest the potential influence of at least two factors. First, the relatively small and uneven sample sizes across investment categories, which vary substantially across utilities, limit the statistical stability of category-level comparisons. Second, recurrence performance is likely influenced by additional factors unrelated to investment in physical upgrades, such as underlying circuit risk characteristics, geographic exposure, or operational conditions and utility decision making, which we could not control for in this analysis. The subsequent sections aim to identify and evaluate some of the potential influences of these additional factors.

We also conducted a duration analysis to evaluate whether investments had a relative impact on reducing de-energization durations. Results across all utilities in Table 3 suggest that investments have little effect on reducing the duration of hours de-energized, although these relationships are not statistically significant. More importantly, however, these resilience investments focus on preventing a de-energization event rather than accelerating re-energization. Therefore, these results should be interpreted as less relevant to the overall study than frequency-based results.

**Table 3:** Duration-based analysis, results for all utilities.

Investment category	Repeat (2018–2022) no. of circuits	Average duration (hours) 2018–2022	Average duration (hours) 2023–2024	Mean change in hours	Median average change in hours	Duration performance ratio, mean hours	Duration performance difference, mean hours	P (Mann Whitney)
No investment	106	40.1	26.0	-14.1	-12.5	1	0	-
Combination	13	47.1	43.6	-3.5	-6.9	0.2	10.6	0.01
Overhead hardening	14	33.7	24.8	-8.8	-9.3	0.6	5.3	0.29
Sectionalizing	33	43.3	32.9	-10.3	-10.4	0.7	3.8	0.18
Undergrounding	2	31.8	22.0	-9.8	-9.8	0.7	4.3	0.65

### Accounting for external wildfire risk factors potentially contributing to circuit de-energizations

We conducted both a macro-level and circuit-level analysis to assess whether these circuit de-energizations could be attributed solely to wildfire risk factors outside the IOUs’ control or if these decisions to de-energize specific circuits were the result of risk factors or decision-making from the IOUs themselves. If de-energizations were primarily the result of external wildfire risks, this would suggest that observed de-energizations reflect exposure to hazard rather than a failure of resilience, introducing complexity into how these events are planned and interpreted. This also speaks to the discretion of each utility and what factors they consider while deciding which circuits to de-energize.

In this section, firstly, we present results using all circuits and non-circuits that have and have not received investment to understand the concentration of circuits generally and to highlight the relationship between potential wildfire risk activity and circuits. Secondly, we conduct circuit-by-circuit analysis and segregate the circuits into different investment classes.

Location quotient results from the High Fire Threat District (HTFD) and de-energized circuit miles suggest that de-energizations from 2018 to 2024 were disproportionately concentrated in areas near or adjacent to HTFD zones (Table 4). This pattern becomes clearer when examining the location quotient, which measures concentration and indicates whether de-energized miles are disproportionately located within high-threat fire zones or surrounding areas. Interestingly, the location quotient is highest in the “core” HTFD zone without buffers. This suggests that while buffer zones account for an increasingly large share of total de-energized miles across the utility, the proportion of overall circuit miles within those buffers exceeds the share of de-energizations occurring there. The location quotient is further supported by the percentage of miles in each area that experienced at least one de-energization. From the perspective of HTFD boundaries, the highest percentages are found within the HTFD zones themselves. Nonetheless, it is notable that by the 10-mile buffer, almost all de-energizations are captured. Conducting the same analysis for circuits affected in 2023 and 2024 produced similar results, suggesting that there was no major difference in the spatial distribution of de-energizations between these years and earlier ones.

**Table 4:** Concentration of circuit miles within HTFD areas, with 2-, 5-, and 10-mile buffers (2018–2024), investment and non-investment.

Utility	HTFD buffer	Category	Percentage of miles across utility with ≥ 1 de-energization	Percentage of miles in area with ≥ 1 de-energization	Location quotient
A	0 miles “Core”	HTFD	47.47	68.58	1.83
		Non-HTFD	52.53	26.55	0.71
B		HTFD	72.58	40.53	3.4
		Non-HTFD	27.42	4.16	0.35
A	2 miles	HTFD	77.91	58.99	1.58
		Non-HTFD	22.09	16.36	0.44
B		HTFD	97.83	22.14	1.86
		Non-HTFD	2.17	0.55	0.05
A	5 miles	HTFD	86.12	51.28	1.37
		Non-HTFD	13.88	14	0.37
B		HTFD	99.72	16.95	1.42
		Non-HTFD	0.28	0.11	0.01
A	10 miles	HTFD	92.12	47.76	1.28
		Non-HTFD	7.88	10.63	0.28
B		HTFD	99.80	14.11	1.18
		Non-HTFD	0.20	0.15	0.01

There are slight differences between Utility A and Utility B, particularly within the HTFD zone (no buffers). Comparing the percentage of miles in an area with at least one de-energization and the location quotient suggests that Utility B has had fewer overall circuits within the HTFD zone de-energized but has a noticeably higher concentration of de-energizations within these zones when they do occur. Overall, the disproportionate concentration of de-energizations for both utilities in HTFD zones and their surrounding buffer areas suggests that the known risk factors used to define the HTFD zones do correspond meaningfully with external factors influencing wildfire risk and utility de-energizations (Table 4).

**Fig. 1:**
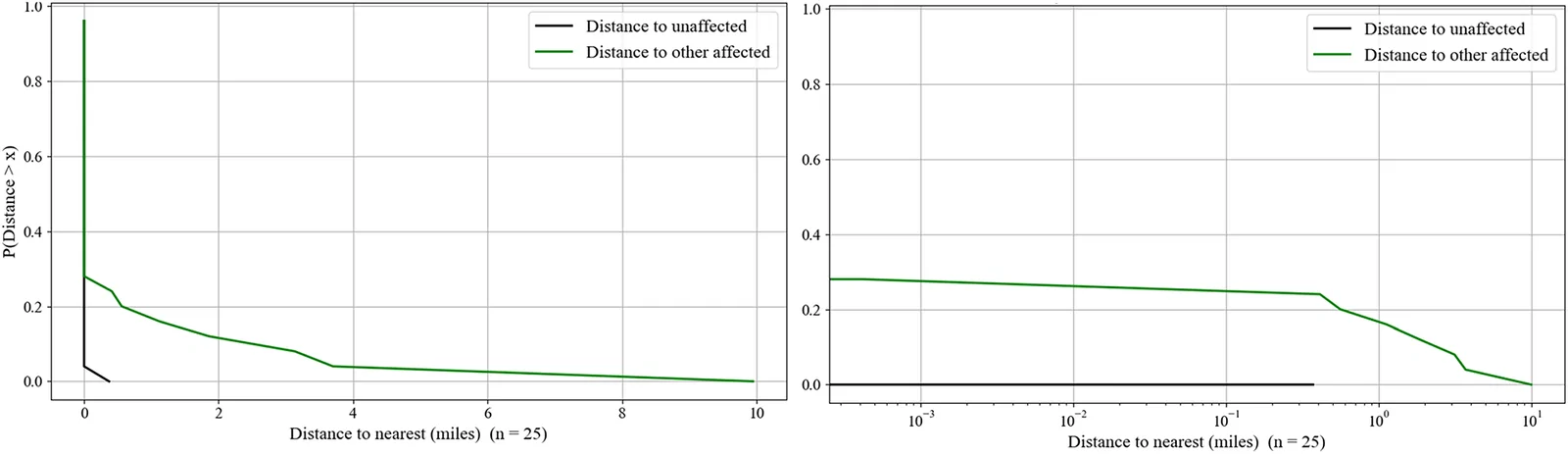
CCDF (normal and log scale) of distance of de-energized circuits that have received investment to the nearest circuit with no de-energization and with de-energization for Utility A, 2023–2024.

**Fig. 2:**
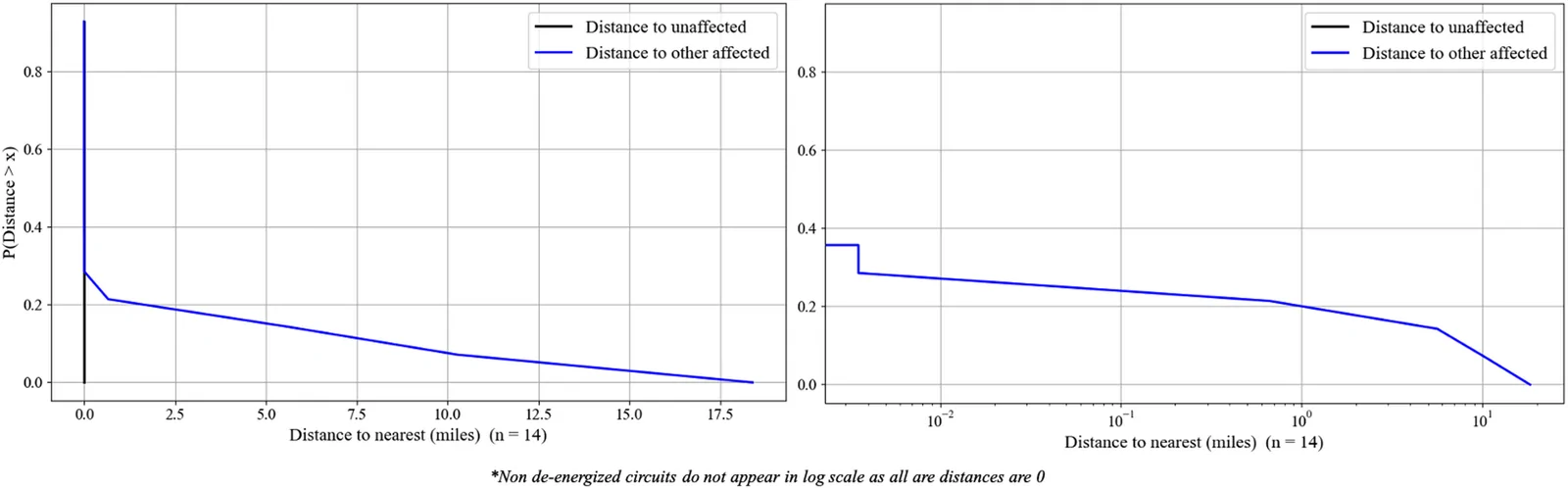
CCDF (normal and log scale) of distance of de-energized circuits that have received investment to the nearest circuit with no de-energization and with de-energization for Utility B, 2023.

Results from the proximity circuit-level analysis suggest that more than half of the de-energized circuits that had received investment were de-energized within a mile of at least one other de-energized circuit, and these are often intermixed with circuits that have not been de-energized (Figs. 1 and 2). A higher concentration of de-energization could point to the influence of risk factors outside the utility’s control (e.g., wind, temperature, dead load vegetation, and moisture). Even with this proximity of de-energized circuits, however, log scale visualizations indicate that the circuit’s proximity to non-de-energized circuits was even closer than its proximity to other de-energized components. Additionally, the tails of these distributions indicate a substantial minority of circuits that are quite isolated from other de-energized circuits, particularly compared to their proximity to non-de-energized circuits.

To strengthen these findings, we used a Wilcoxon statistical test to determine the probability that these circuits were closer to a non-de-energized circuit than to a de-energized circuit, with the null hypothesis being that there was no substantive difference. Results from Wilcoxon statistical tests support this finding, with p-values well below 0.05 across all scenarios except for circuits that received investment from Utility B. These circuits may appear less distinct, possibly because many of these circuits are equally close to both de-energized and non-de-energized circuits (i.e., they are touching both types). Additionally, the smaller sample size for B’s investment circuits may skew the results, which remain low (0.13) but not below the 0.05 threshold. Effect size calculations using biserial rank also indicate a strong effect difference between the proximity of de-energized circuits to non-de-energized circuits, which is likely driven by the tails of the CCDF results. A combination of significant p-values and large effect sizes suggests wildfire risk inputs outside the utilities’ control (e.g., wind, temperature, dead load vegetation, and moisture) do not fully explain their de-energization decisions. Rather, other circuit-specific (e.g., condition) or operational factors (critical load designation, high risk areas) are likely influencing these outcomes, which could be clarified with more data and additional study (Table 5).

**Table 5:** Likelihood that a de-energized circuit is more proximate to a non-de-energized circuit than the nearest de-energized circuit, by utility and investment type.

Utility	De-energized circuit type	Wilcoxon p	Biserial rank	Number of circuits
Utility A	Investment	0.0076	-0.91	25
Utility A	Overhead hardening	0.0339	-1.00	8
Utility A	Sectionalization	0.0125	-0.89	23
Utility A	Undergrounding	0.0899	-1.00	3
Utility A	No investment	4.57E-11	-0.93	82
Utility A	All	3.35E-12	-0.92	107
Utility B	Investment	0.0136	-1.00	14
Utility B	Overhead hardening	0.0136	-1	14
Utility B	Sectionalization	0.0899	-1	3
Utility B	No investment	0.0004	-0.98	37
Utility B	All	3.44E-05	-0.99	51

The sum of circuits by investment type is greater than the total number of circuits since 6 circuits and 3 circuits in Utilities A and B respectively received a combination of investments. We conducted a separate analysis for these circuits and derived similar results.

Results from the CCDFs at normal and logarithmic scales of distance also suggest a minimal difference between circuits with investment as opposed to circuits without investment, which was supported by additional statistical analysis in Table 5 (note, p values for two of the investments exceeded the standard threshold of 0.05, which along with limited data for all investment classes, should caution an interpretation of the results). For this reason, we did not show CCDFs that aggregate between circuits with investment and without investment. The relative similarity in proximity between de-energized and non-de-energized circuits should not necessarily suggest that these circuits are located in similar parts of the IOU’s served, as this analysis does not evaluate circuit density or the number of proximate circuits, but rather the distance to the closest proximate circuit. With this said, however, results showing minimal differences between circuits that received investment and those that did not receive investment support the analysis that there seem to be minimal discernible performance of de-energized circuits that had and had not received investment.

## Discussion and conclusion

Results indicate that physical infrastructure investments from 2023 to 2024 seem to have either not reduced the number of de-energizations at all or were not sufficiently calibrated to account for the higher risk levels in these areas. Current grid hardening strategies do not seem to have reduced de-energizations in high-risk areas. These findings shed new light on PSPS research, which has largely relied on simulation modeling^16–19^, by providing an empirical case to evaluate the investments to reduce PSPS. The results inform research that questions PSPS as an appropriate way to mitigate wildfire risk^18,19^ by highlighting that current strategies to reduce de-energizations may not have been effective when evaluated in isolation. This study does not necessarily share the conclusions of previous work disputing PSPS overall, since it does not assess the tradeoff of de-energizations relative to wildfire risks. Instead, it addresses the specific period under analysis by seeking to isolate these investments as much as possible. The results also contribute to broader work on infrastructure resilience by offering one of the few empirical evaluations of physical investments in electricity systems^21–23^.

Geospatial outputs from the second results section are concentrated in or near HTFDs, which aligns with some of the grey and academic literature on where PSPS events were likely to be concentrated^18,19,24^. This initially suggests that de-energizations may be the IOUs’ response to external wildfire risk factors. To test this hypothesis, we conducted a proximity analysis ​to isolate the role of external wildfire risk factors in circuit-level de-energizations, which is novel for PSPS research. Our preliminary results reveal that de-energized circuits are almost always located closer to a non-de-energized circuit than to the nearest other de-energized circuit—and in close proximity in absolute distance—irrespective of investment type, suggesting that external wildfire risk alone does not account for the de-energizations. The results both advance and complicate existing literature on PSPS events. On one hand, prior work has suggested that PSPS events would be concentrated in HFTD areas^18,19,24^, which these results demonstrate. We advance the specificity of the role of wildfire risk by indicating that wildfire risk alone does not fully explain why specific circuits were de-energized.

Utilities may benefit from furthering their existing wildfire mitigation strategies by continuing to consider a broader and more dynamic set of considerations. These include engineering factors, regulatory frameworks, wildfire risk modeling, liability exposure, and per-customer impacts over longer time horizons. Building on current practices in this way could support more efficient planning, strengthen institutional learning, and improve the ability to estimate the long-term impacts of de-energization decisions in current dollars. Given the increasing wildfire risk in parts of the U.S. associated with changing climate conditions, it may also be useful to consider how existing mitigation strategies perform over time. Evaluating the longer-term net effects of different approaches could help inform discussions about how grid infrastructure evolves, rather than relying solely on incremental hardening measures. Such analysis may provide additional insight into how utilities can balance reliability, risk reduction, and system planning objectives.

The outputs from our limited sample also reflect that these investments might have diminished returns relative to other components to make these de-energization decisions, such as monitoring, re-generation protocol, and other non-hardening measures. Diminished returns warrant the question of whether these resources are being optimally allocated when considering their resilience to PSPS. From an energy economics perspective, the persistence of relatively high PSPS recurrence rates and limited reductions in de-energization frequency suggests that these investments may yield modest improvements when evaluated against the customer impacts they are intended to avoid, despite high capital costs. These considerations should be taken into account in the broader debate about wildfire risk and the potential energy efficiency benefits of these investments. Nonetheless, the findings also merit discussion of the benefits of alternative adaptive investments and policies.

The IOUs and policymakers may wish to emphasize alternative resilience strategies to address these empirical gaps in investment efficacy, particularly strategies that allow for localized backup generation and distribution system enhancements at the circuit level (e.g., a microgrid) or at the level of the individual consumer (e.g., a backup generator). Some sources (including Commentator B) indicated that microgrids may be a useful tool in helping minimize the negative impact of PSPS events, as these grid de-energizations can lead to extended power interruptions across IOU service territories^22^. In one of the early scoping memos of rulemaking R.19-09-009 (initiated as a result of Senate Bill 1339), the CPUC noted that microgrids are one of the resilience strategies introduced in the state to reduce the impacts of PSPS de-energizations as well as other outage events^23^. If the wildfire risk mostly threatens the distribution network, however, a microgrid or similar technology may not be an appropriate solution, since these technologies do not necessarily mitigate why the power was de-energized in the first place (i.e., because the utility had concerns with the actual distribution system, such as the lines, conductors, etc., on the specific circuit). It is also important to note that these alternative technologies may introduce different types of risk to the grid^25^. In this case, pursuing hardening measures with more demonstrated effectiveness or providing resources through state-sponsored programs such as the Self-Generation Incentive Program^26^ may be more effective.

Further research could help identify more precisely which circuits have experienced de-energizations and to link these patterns with demographic characteristics. An analysis that linked circuit-level infrastructure to socio-spatial data was beyond the scope of this study due to limitations in data availability and privacy protections. From a systems perspective, adding this spatial angle could expand the analysis into equity and long-term wildfire mitigation strategies and see if these investments are merely shifting the risk instead of eliminating it.

Future work could also examine how PSPS events, particularly those affecting the most frequently de-energized circuits (e.g., those in Utility B’s territory), intersect with broader risks such as insurance withdrawal or other cascading effects of wildfire exposure. Such a compounding risk study could offer a more complete picture of resilience, from an energy systems perspective as well as a local vulnerability and capacity perspective. Additional utility data or expert interviews involved in de-energization decisions may shed new light on the specific contributing factors, which are not easily discernible from publicly available data. This type of analysis could advance electricity resilience research more broadly, particularly by highlighting how the role of human decision-making and other non-technical components can influence whether specific circuits are de-energized^2^. The results of this study would benefit from a significantly larger body of investments to analyze. All three IOUs have pledged to undertake significant additional investments to minimize wildfire risk and PSPS, particularly undergrounding^27–29^. We recommend revisiting this analysis and potentially incorporating some of the future research directions raised here.

## Methods

We address the question of whether circuit-level investments to minimize de-energizations from PSPS events have demonstrably reduced the frequency or severity of de-energizations using two approaches, which are presented in two separate results sections. First, we compare the frequency of PSPS de-energizations in 2023–2024 on circuits that received infrastructure upgrades between 2018 and 2022 to those that did not receive upgrades. Second, we conduct a geospatial analysis of de-energization patterns to assess how the location of de-energized circuits corresponds to known wildfire risk areas and their closest neighboring de-energized and non-de-energized circuits. The geospatial analysis helps determine whether PSPS events during this period were primarily a response to wildfire conditions outside the IOUs’ control (e.g., high wind speed, temperature, dead fuel) or whether the spatial distribution also implies the influence of internal factors (e.g., utility decision-making processes or circuit-specific characteristics) on de-energizations. We conducted this analysis by examining investments both as a combined category and more disaggregated by investment type and by utility. The benefits and limitations of each approach are discussed in the following sections.

### Overview of PSPS events and existing research

Although California regulators formally recognized the use of PSPS events in 2012, their mandated implementation across IOUs did not begin until 2018, following a series of significant wildfires, some of which were attributed to ignitions from the electricity grid^7^. The number and frequency of de-energizations have increased substantially since 2018, particularly among the state’s three largest investor-owned utilities (IOUs). Together, these IOU serve over 12 million customers and cover more than 75% of California’s land area^30–32^.

### Data collection

Almost all this work involved analytic research conducted using publicly available websites. During the course of the study, we interviewed two experts not to generate additional results, but to verify that the data used was the most accurate available and to confirm that no relevant data had been overlooked. Only comments from two commentators (A and B) are featured in this paper. These conversations also provided general insights into how IOUs conduct PSPS events, helping to ensure that our arguments were appropriately contextualized. Although this paper does not aim to produce findings from semi-structured interviews, the interviews served as complementary checks on our data sources and interpretations. In keeping with ethical standards, the research team followed an ethics review process consistent with EU 2018 data protection regulations and IRB protocols.

#### De-energizations by circuit, base data (2018–2024)

The California Public Utilities Commission (CPUC)^33^ provides an excel file on its website containing PSPS data, which was collected and used for this analysis. For further validation, the data was also vetted using publicly available information on the CPUC’s dashboard^34^ as well as PSPS reports^35–37^. We then cleaned the data to prepare it for analysis and disaggregated it at the circuit level. It is important to note that transmission-level de-energization data was removed from the dataset as it lacked sufficient detail to distinguish de-energizations on transmission lines and circuit-level de-energizations caused by upstream transmission de-energizations, which would have led to double counting. Due to these data limitations, it also was difficult to determine which sections of the transmission system were affected, further complicating efforts to identify which customers may have experienced de-energizations. Similarly, the way utilities reported de-energizations made it nearly impossible to determine which specific portions of a sub-circuit were affected, making the full circuit the smallest reliably identifiable unit of analysis for this study.

Lastly, to minimize error probabilities (including over- and underestimation of results) due to inconsistent circuit names, circuit names were also matched with other datasets to ensure consistency. All data sources were triangulated to ensure consistency prior to matching circuit names across de-energization records. For two of these utilities, ICA (integration capacity assessment) data was used as the primary source of matching. The other utility required further clearance to use ICA, which we were unable to obtain due to time, resources, and other constraints.

#### Investment data

The investment analysis uses all collected data on de-energizations by circuit, along with additional information on investments and factors to contextualize the potential role that investments in physical infrastructure may have played in changing de-energizations over time. Similar to PSPS data, all information on investments in physical infrastructure to reduce the impact of PSPS events was collected from mandatory reporting by the IOUs on areas of greatest impact from PSPS events and enacted or planned measures to reduce these impacts. Information on investments at the circuit level was collected from utilities’ wildfire risk plans, which constituted the bulk of the investment data analyzed^13,38,39^. Commentators confirmed that these plans were the most comprehensive during the collection phase (Commentators A and B). These data were cross-checked against investment plans on facility websites, along with other declarations and testimony regarding investments^27,40,41^.

A scan of websites and public documents similarly indicated that wildfire plans offered the most complete accounting of investment activity for PSPS events, as no other major dataset was found beyond these plans, and the limited information found outside these plans supported the information outlined in these plans. Expert input similarity suggested that no better data sources existed. Only investments that had reached the final stage of completion were included in the analysis. No circuits with planned investments were included. Investments in circuit-level infrastructure were grouped by intervention.

When analyzing the role of utility investments to improve circuit performance, it is important to disaggregate these investment types to distinguish between the extent of their impact on circuit improvements. The CPUC categorizes broad utility measures to minimize wildfire ignition risk^42^. These include vegetation management, system hardening, grid topology improvements, inspection and maintenance, situational awareness, and community engagement. Based on these broad categories, we classified investment categories (as shown in the Approach and Methods Table) for further analysis. For clarity and exhaustiveness, we put relevant activities within each of the three categories based on CPUC’s Wildfire Mitigation Plan (Sect. 7.3.2).

**Table:** Approach and methods table – utility grid investment categories.

Category	Activities	Tag
Overhead hardening	Covering conductors, upgrading equipment, vegetation management, circuit breaker maintenance, capacitor maintenance, T&D pole replacement and reinforcement, system automation equipment, pole loading infrastructure hardening, expulsion fuse replacement, crossarm upgrades, insulated hardware, Rapid Earth Fault Current Limiters (REFCLs)	OH
Undergrounding	Partial or complete undergrounding power lines, circuits, equipment, feeders	U
Sectionalizing	Making grid topology improvements (such as installation and operation of electrical equipment to sectionalize or island portions of the grid), expulsion fuse replacement, SCADA-controlled reclosers, automated sectionalizing switches, branch line fuses, load transfer switches, islandable microgrid points	S

Circuits tagged as ‘OH’, ‘Overhead hardening and activities’ include upgrading equipment, circuit breaker maintenance, transmission and distribution line repairs, vegetation management, infrastructure hardening and more. Secondly, ‘undergrounding’ is tagged as ‘U’ and includes activities such as partial or complete undergrounding. Lastly, ‘sectionalizing’ tagged as ‘S’ includes making grid topology improvements, installing equipment to sectionalize, load transfer switches, islandable microgrid points, and related upgrades^43^. Based on these investment archetypes, utility activities under the target period of analysis were systematically coded. Basic summary statistics (in the Investment Activities Summary Statistics Table) of these investment activities indicate that over 40% of the total investment activities under analysis were based on sectionalization. Around 29% of the total investment activities were overhead and less than 4% accounted for undergrounding. Over 27% of activities were combinations involving two or more of these activities.

**Table:** Approach and methods table – investment activities summary statistics.

Tag		Count of measures		%	
OH		61		28.77	
OH, U		9		4.25	
S		85		40.09	
S, OH		28		13.21	
S, OH, U		15		7.08	
S, U		6		2.83	
U		8		3.77	
Total		212		-	

#### Geospatial data

The geospatial data was collected by verifying name matches and linking them to specific circuits using the ICA data, which was also used to support circuit-level matching in the de-energization analysis (Citations for all data sources are provided in the accompanying data table. Specific citations identifying the two utilities used in this analysis are not included here in order to better obfuscate utility-level results). For this reason, only the two IOUs with available ICA data were included in the second part of the analysis.

### Data processing and analysis

#### De-energizations

Using a frequency-based approach, we calculated circuit-level disruptions by treating any de-energization events that began simultaneously and occurred on the same circuit as part of a single event, before linking these circuits to investment and geospatial data. Our approach aligns with how utilities have measured de-energizations at the circuit level (see^44^, for example), with one addition. We treat differences in re-energization at the circuit and/or sub-circuit level as two distinct events. For instance, if one re-energization occurred several hours or days after a prior re-energization, we classify them as separate events at the circuit level. We made this distinction to highlight differences in the duration of de-energization as a proxy for hardship on specific circuits or clustered sub-circuits that experienced longer disruption times. As a post-processing phase, the IOUs were randomly assigned pseudonyms “Utility A”, “Utility B”, and “Utility C” in the results to better obfuscate the specific circuits that experienced higher levels or frequency of de-energization events within each IOU.

#### Investment data

After tagging circuits by investment class (4.2.2), we defined the baseline cohort (*B*) as all circuits that were de-energized at least once during 2018–2022. We then conducted an observational analysis to evaluate the performance of these same circuits during the subsequent period from 2023 to 2024 (*R*) to measure how often de-energizations recurred and to compare recurrence performance across investment categories.

##### Frequency-based analysis

To set up the observational analysis, we disaggregated circuits in both periods into their respective investment classes. This allowed direct comparison of recurrence behavior between the baseline period (2018 to 2022) and the observation period (2023 to 2024). Circuits classified as having received no investment were classified as part of category . Circuits classified as having received some form of investment were classified as part of category *k*.

Several circuits received more than one type of investment. To account for this, we conducted two complementary analyses. In the first analysis, each circuit was treated as a unique observation and assigned to a single mutually exclusive category. Circuits that received multiple investment types were classified as a separate “combined investment” category (*C*). This ensured that each circuit was counted only once and allowed comparison across distinct investment classifications. In the second analysis, we evaluated each investment category independently to assess whether receiving a specific type of investment was associated with improved recurrence performance. In this case, circuits that received multiple types of investment were included in each applicable investment category (i.e., potential for double-counting). This approach allowed evaluation of the performance of individual investment types, but does not represent mutually exclusive circuit groups. In both analyses, we assumed that investment ($\:k,\lambda\:$) status remained fixed during the study period (see limitations section). All other aspects of the analysis were identical. Our notation for these equations is below:

$$\:B=\{\mathrm{c}\in\:U:\exists\:\mathrm{\:de-energization\:event\:for\:circuit\:(}c)\mathrm{\:during\:}{T}_{0}\}$$

$$\:R=\{\mathrm{c}\in\:B:\exists\:\mathrm{\:de-energization\:event\:for\:circuit\:(}c)\mathrm{\:during\:}{T}_{1}\}$$

Where * U* $\:\in\:$ *circuits in Utility A*,* Utility B*,* Utility C*,* or all Utilities combined*

$$\:{T}_{0}=2018\:\mathrm{-\:}2022$$

$$\:{T}_{1}=2023\:\mathrm{-\:}2024$$

$$\:g\left(c\right)\in\:\{k,{\uplambda\:}\}$$

$$\:k\:\in\:\{OH,S,U,C\},\:\hspace{1em}\hspace{1em}{\uplambda\:}=\:o$$

$$\:{B}_{\lambda\:,k}=\{c\in\:B:g\left(c\right)=k,\lambda\:\}$$

$$\:{R}_{\lambda\:,k}=\{c\in\:R:g\left(c\right)=k,\lambda\:\}$$

We evaluated recurrence rate and observed share of de-energizations to determine how often circuits in each investment category experienced repeat de-energizations and whether those repeats occurred more or less often than expected. We defined expectation as the recurrence distribution that would be expected if recurrence occurred proportionally to the baseline distribution of circuits across investment categories during 2018–2022. We set the recurrence rate for each investment or non-investment category (*k*,* λ*) as the fraction of circuits in that category that were de-energized again in 2023–2024. We used the recurrence rate as a measure for circuits in each category that experienced repeat de-energizations after the baseline period. We also compared the observed share of repeat de-energizations to the expected share based on the baseline period. The observed share is the fraction of circuits in each category among those that experienced repeat de-energizations in 2023–2024. Comparing observed and expected shares helps show whether circuits in a given investment category experienced more or fewer repeat de-energizations than would be expected based on their original share. These ratios are expressed below as:

$${\mathrm{Recurrence~rate~}} = \frac{{\left| {R_{{k,\lambda }} } \right|}}{{\left| {B_{{k,\lambda }} } \right|}}$$

Observed share of de-energizations over expected share of de-energizations = $\:\frac{\left|{R}_{k,\lambda\:}\right|}{\left|R\right|}/\frac{\left|{B}_{k,\lambda\:}\right|}{\left|B\right|}$.

Building on the recurrence rate and observed share of de-energizations, we compared recurrence performance directly between investment and non-investment circuits to determine whether investment was associated with improved outcomes. This was done by adapting statistical measures of probability from risk analysis—risk ratio and risk difference^45^—which we refer to as recurrence performance ratio and recurrence performance difference since these ratios relate to observed performance. The recurrence performance ratio compares the recurrence rate of circuits in investment category (*k*) to the recurrence rate of circuits in the reference category of no investment (λ). A value less than 1 indicates that circuits in the investment category experienced lower recurrence in 2023–2024 relative to the no-investment group, while a value greater than 1 indicates higher recurrence. The recurrence performance difference provides an absolute measure of recurrence performance by subtracting the recurrence rate of the reference category (λ) from that of the investment category (k). A negative value indicates improved recurrence performance relative to the reference group, while a positive value indicates a comparatively worse recurrence performance. These equations are expressed below as:

$${\mathrm{Recurrence~performance~ratio~}} = \frac{{\left| {R_{k} } \right|}}{{\left| {B_{k} } \right|}}/\frac{{\left| {R_{\lambda } } \right|}}{{\left| {B_{\lambda } } \right|}}$$

$${\mathrm{Recurrence~performance~difference~}} = \frac{{\left| {R_{k} } \right|}}{{\left| {B_{k} } \right|}} - \frac{{\left| {R_{\lambda } } \right|}}{{\left| {B_{\lambda } } \right|}}$$

We calculated a p-value for statistical significance using Fisher’s Exact Test. Fisher’s Exact Test was selected because it provides an exact statistical inference method appropriate for small sample sizes and uneven group distributions (see investment categories numerically)^46,47^. In this case, we evaluated whether the observed recurrence rates differ significantly between each investment category (*k*) and the reference category of no investment (λ) using the contingency structure defined by baseline and recurring circuits. We denoted Fisher exact test using the conventional notation, (*i; a*,* b*,* c*,* d*,* n*) for readability, with the appropriate values specified for each of these variables in this case. For our null hypothesis ($\:{H}_{0})$, we proposed that investment status does not affect the recurrence probability of circuit de-energizations in 2023–2024. These equations are expressed below as:

$${\text{p }}(Fisher's~exact)_{{k,\lambda }} = \sum\nolimits_{{a^{\prime}:P\left( {a^{\prime},b^{\prime},c^{\prime},d^{\prime}} \right) \le P\left( {a,b,c,d} \right)}} {\frac{{\left( {a^{\prime} + b^{\prime}} \right)!\left( {c^{\prime} + d^{\prime}} \right)!\left( {a^{\prime} + c^{\prime}} \right)!\left( {b^{\prime} + d^{\prime}} \right)!}}{{a^{\prime}!\,b^{\prime}!\,c^{\prime}!\,d^{\prime}!\,n!}}}$$

Where

$$\:a=\left|{R}_{k,\lambda\:}\right|,\hspace{1em}b=\left|{B}_{k,\lambda\:}\right|-\left|{R}_{k,\lambda\:}\right|,\hspace{1em}c=\left|R\right|-\left|{R}_{k,\lambda\:}\right|,\:\:\mathrm{d}=\left|{B}_{k,\lambda\:}\right|-\left|{B}_{k,\lambda\:}\right|-\left(\left|R\right|-\left|{R}_{k,\lambda\:}\right|\right),\hspace{1em}n=a+b+c+d=\left|B\right|$$

$$\:{H}_{0}:\:\frac{\left|{R}_{k}\right|}{\left|{B}_{k}\right|}=\frac{\left|{R}_{\lambda\:}\right|}{\left|{B}_{\lambda\:}\right|}$$

##### Duration-based analysis

To complement the frequency-based outage analysis and to begin addressing outage duration, we examined the average number of de-energization hours experienced by circuits. We first restricted the analysis to circuits that experienced de-energizations in both time periods, denoted R (see previous section), to isolate changes in duration between the baseline and observation periods.

$$\:R=\{c\in\:U:\exists\:\mathrm{\:de-energization\:event\:for\:circuit\:}c\mathrm{\:during\:}{T}_{0}\wedge\:\:\exists\:\mathrm{\:de-energization\:event\:for\:circuit\:}c\mathrm{\:during\:}{T}_{1}\}$$

We defined *h(e)* as the number of de-energized hours for a circuit during a PSPS event *e*. We then aggregated these values at the circuit level. For each circuit (*c*), the total number of de-energized hours during a given time period T was calculated as the sum of the hours (*H(c)*) across all PSPS events affecting that circuit during the period. The time periods *T* remained consistent with those defined previously, with $\:{T}_{0}\:$representing 2018–2022 and $\:{T}_{1}$ representing 2023–2024. We standardized the comparison across circuits and time periods by calculating the average outage duration $\:\stackrel{-}{{(H}_{k,\lambda\:,T}})\:$by dividing the total outage hours across circuit investment/non-investment classes by the number of PSPS events affecting that class within the corresponding period *T* (with $\:\lambda\:$ and *k* denoting the same investment/non-investment classes as the previous section).

$$\:H\left(c,T\right)={\sum\:}_{d\in\:\mathcal{E}\left(c,T\right)}h\left(e\right)$$

$$\:{\Delta\:}\stackrel{-}{{H}_{k,\lambda\:,T}}=\frac{1}{\left|{R}_{k,\lambda\:}\right|}{\sum\:}_{c\in\:{R}_{k,\lambda\:}}H\left(c,T\right)$$

We conducted several tests to evaluate differences in outage duration across circuit classifications. We examined the average duration and the change in average duration between $\:{T}_{0}\:$and $\:{T}_{1}\:$ to determine whether circuits with the same types of investments exhibited reductions in outage duration. This comparison provided a baseline reference for identifying notable changes in circuit performance over time.

$$\:{\Delta\:}\stackrel{-}{{H}_{k,\lambda\:,T}}=\frac{1}{\left|{R}_{k,\lambda\:}\right|}{\sum\:}_{c\in\:{R}_{k,\lambda\:,}}\left(H\left(c,{T}_{1}\right)-H\left(c,{T}_{0}\right)\right)$$

We also conducted a duration performance ratio, where we used circuits that had not received investment as the reference group to maintain consistency with the previous section. We also calculated the duration performance difference, which is defined as the absolute difference between the average duration of circuits that received investment and the average duration of circuits that did not. Comparing both measures allowed us to evaluate both the proportional difference in duration and the absolute magnitude of the difference between categories. This distinction is useful because proportional changes can sometimes overstate practical impacts. For example, a large proportional change may represent only a small change in the actual number of outage hours experienced by customers.

$$Duration~performance~ratio~ = ~\frac{{\Delta \overline{{H_{k} }} }}{{\Delta \overline{{H_{\lambda } }} }}$$

$$Duration~performance~difference~ = \Delta \overline{{H_{k} }} ~ - ~\Delta \overline{{H_{\lambda } }}$$

We used a Mann–Whitney U test to evaluate statistical significance. Unlike the recurrence analysis, which relied on Fisher’s Exact Test due to the categorical structure of the contingency table, the duration analysis compares continuous outage duration values across circuit classifications. The no investment category (λ) was selected as the reference category for an analysis. The Mann–Whitney U test was selected because it is a nonparametric test that does not assume normally distributed data and is appropriate for comparing two independent samples with potentially uneven sample sizes. This test is also useful because de-energization duration data are typically right skewed as they are bounded by a floor of zero and may contain extreme events. For the null hypothesis $\:{(H}_{0})$, we posit that the distribution of outage durations for circuits that received investment is the same as the distribution of outage durations for circuits that did not receive investment.

$$\:U=\mathrm{min}\left({U}_{k},{U}_{\lambda\:}\right)$$

$$\:{\mu\:}_{U}=\frac{{n}_{k}{n}_{\lambda\:}}{2}$$

$$\:{\sigma\:}_{U}=\sqrt{\frac{{n}_{k}{n}_{\lambda\:}\left({n}_{k}+{n}_{\lambda\:}+1\right)}{12}}$$

$$\:{H}_{0}:{F}_{k}\left(h\right)={F}_{\lambda\:}\left(h\right)$$

#### Geospatial data

Both the large-scale geospatial analysis of circuits within High Fire Threat District (HTFD) areas and the more granular circuit-level proximity analysis use the same underlying circuit data, which were compiled using ICA datasets. We tested the overlay of HTFDs by taking the individual ICA circuits and overlaying them with the HTFD areas^48^. To improve performance and reduce computational cost, we developed two data frames: one containing all circuits with at least one de-energization (based on the criteria outlined in the general data circuit processing and analysis), and another containing all circuits regardless of de-energization status. Unlike the previous section, we evaluated investment status using a binary classification of investment (Y) and no investment (N). This approach was used in part due to the limited number of investments within individual categories, which made more granular concentration-based comparisons less statistically meaningful. The binary classification allowed for a more stable comparison of recurrence performance between circuits that had received any investment and those that had not. We parallelized the processing for the two utilities to enhance computational speed and also included buffers of 2, 5, and 10 miles from the HTFD area to assess what percentage of circuit miles were located within these areas. We did not disaggregate HTFD zones into Tier 2 and Tier 3 since buffering these zones would naturally expand Tier 2 zones at significantly disproportionate rates compared to Tier 3 zones. Instead, we processed the data as HTFD with an optional buffer and treated the remaining area as the rest of the utility service territory.

The results were presented in percentages rather than total miles to better obscure the identity of the utilities. For this reason, the results show the percentage of affected circuit miles as a percentage of circuit miles in each zone (both HTFD and non-HTFD) as a percentage of all de-energized circuits. This was done to provide an understanding of where most of these de-energizations were occurring or had occurred. In addition, we applied a location quotient (LQ; see below) to account for disparities in the number of circuit miles in each zone, which could bias results (for example, if 97% of all de-energized circuit miles were located in Zone A but Zone A also contained 97% of all circuits, which would be proportional). Location quotients are commonly used to assess concentrations in economics, like industry concentrations^49,50^. Since this work involves comparing percentages, they are well suited to help determine whether de-energizations are more heavily concentrated in certain areas, such as within HTFD zones or in areas outside of them. This adjustment helps normalize the distribution of circuit miles across the zones.

$$\:L{Q}_{B}^{\:HTFD}=\frac{\mathrm{Deenergized\:}{\mathrm{Miles}}_{\mathrm{HTFD}+B}/\mathrm{Total\:}{\mathrm{Miles}}_{\mathrm{HTFD}+B}}{\mathrm{Deenergized\:}{\mathrm{Miles}}_{\mathrm{Non-HTFD}-B}/\mathrm{Total\:}{\mathrm{Miles}}_{\mathrm{Non-HTFD}-B}}$$

$$\:L{Q}_{B}^{Non\:HTFD}=\frac{\mathrm{Deenergized\:}{\mathrm{Miles}}_{\mathrm{Non-HTFD}-B}/\mathrm{Total\:}{\mathrm{Miles}}_{\mathrm{Non-HTFD}-B}}{\mathrm{Deenergized\:}{\mathrm{Miles}}_{\mathrm{HTFD}+B}/\mathrm{Total\:}{\mathrm{Miles}}_{\mathrm{HTFD}+B}}$$

Where B = Buffer distances ∈ {0, 2, 5, 10}.

For the circuit-level proximity analysis, we calculated the shortest distance between each circuit and the nearest de-energized circuit using a minimum Euclidean distance. This method did not account for terrain or other spatial features since the goal of this analysis was not to identify the specific factors driving de-energization decisions, but rather to examine whether de-energized circuits frequently occurred in very close proximity to non-de-energized ones.

Results of this distance were first calculated visually using complementary cumulative distribution functions (CCDF; = P(X ≤ x)). We conducted this experiment in alignment with the previous two sections’ structure. We first conducted a binary analysis in which circuits were classified as having received investment (Y) or no investment (N). We also conducted separate analyses for each individual investment class to evaluate recurrence performance across specific investment types (OH, S, U, C; see 4.2.2). We conducted both analyses because the circuit-level granularity of the data allowed for direct comparison across specific investment types. However, the limited number of circuits in several investment categories reduced the statistical reliability of category-level comparisons. The binary classification provided a more stable basis for evaluating recurrence performance between circuits that received investment and those that did not.

These functions were used to illustrate the proximity of de-energized circuits to their nearest de-energized and non-de-energized counterparts. In this case, the x-axis represents distance from the nearest other circuit in miles, while the y-axis shows the proportion of circuits that exceed that distance threshold. For example, x = 2.5 and y = 0.40 on the CCDF would indicate that 40% of de-energized circuits are more than 2.5 miles away from the nearest circuit of the specified type. Results did not demonstrate a significant difference between circuits that were de-energized during 2023 and 2024 and had received investment versus those that had not, which was supported by the results of the statistics. Therefore, we present the CCDFs for all de-energized circuits collectively in normal and log scale (x-axis), rather than disaggregating investment status. We display a side-by-side comparison of both the normal and logarithmic scales to highlight the heavy tail of the distance distribution (normal scale) while also capturing small differences at higher proportions (i.e., as y approaches 1) with the log scale axis.

To strengthen the findings in the CCDF visual analysis, we used a Wilcoxon statistical test, which is well established for evaluating differences in paired data^51,52^, to determine the probability that these circuits were closer to a non-de-energized circuit than to a de-energized circuit, with the null hypothesis being that there was no substantive difference. To supplement this analysis with a proxy for “effect size,” we also calculated the rank biserial to evaluate the magnitude of the correlation between distances to de-energized and not de-energized circuits^51^. This application of the rank biserial test frames the null hypothesis as a positive or near-zero effect size within the range [0, 1], based on the assumption that the distance from a de-energized circuit to its nearest non-de-energized circuit is not consistently greater than the distance to the nearest other de-energized circuit. By contrast, values closest to -1 would demonstrate a strong correlation with the proximity of de-energized circuits to non-de-energized circuits compared to the proximity of these circuits to other de-energized circuits.

### Limitations

Most of the limitations in this paper stem from the relatively limited availability of investment data, which constrains the statistical power of the analysis and makes it difficult to draw definitive conclusions about the direction and magnitude of the findings. Additional limitations arise from assumptions regarding underlying risk profiles and other structural characteristics that shape the relationship between resilience investments and circuit performance. More comprehensive data on exposure, system configuration, and resilience-related attributes would likely improve interpretability and help clarify the mechanisms underlying the observed results.

It can be difficult in these reports to disaggregate which disruptions resulted from a technical issue, a hazard event, or another impact. This issue is further compounded by the fact that no comprehensive dataset appears to exist on circuit-level investments for all disruption types or the rationale behind their inclusion (this assertion refers to a comprehensive dataset for disruptions in general, not necessarily for worst-performing circuits or other prominent disruptions, which IOUs are mandated to report publicly). The lack of data makes it nearly impossible to extract a comprehensive understanding of investments in the physical components of electricity systems at the grid and circuit levels of resilience as defined in the context of this study. Given these limitations, we focused on using 2023–2024 data to test whether the proportion of circuits de-energized had changed substantially. This may indicate the effectiveness of physical infrastructure investments in reducing de-energization events, but not necessarily in reducing average hours of de-energized or average customers impacted.

During this analysis, we assumed that circuits categorized as having no physical investment did not receive any new investment during 2023–2024, which introduces potential bi-directional This assumption was necessary because comprehensive data on investments implemented during 2023–2024 did not seem to be publicly available at the time the experiment was conducted. As a result, circuits classified as having no physical investment were assumed not to receive new investment during the follow-up period. This introduces potential misclassification bias. The direction of that bias is theoretically ambiguous. If newly invested circuits experienced fewer de-energizations, their continued inclusion in the reference group would artificially improve the apparent performance of the no-investment category, making the investment categories appear relatively worse. Conversely, if newly invested circuits continued to experience de-energizations, their inclusion in the reference group would increase the recurrence rate of the reference category, dampening the apparent difference between investment and non-investment circuits.

It is also important to recognize that the investment classifications used here are necessarily coarse and overlook variation in some risk factors inherent to differences across circuits. Substantial heterogeneity may exist within each investment category in terms of scope, configuration, implementation quality, and local operating conditions. For instance, distribution lines (as well as smaller circuits on them) vary in length, voltage, terrain they cover, topology, and their specific wildfire risk exposure. Treating all these circuits as homogeneous was a simplicity choice, but it is important to mention this as a major limitation. Longer lines will inherently face higher wildfire risk, and the number of customers a line serves also implies the extent of impact its outage will have. Aggregating these risks can create a bias and result in potential under or over estimation of outputs. Similarly, results were also not controlled for customer base type or density. These factors can determine the impact of outage based on whether the line serves critical customers or not. This can also, theoretically, lead to oversimplification of resilience outputs and implications of de-energizations. A full assessment of those differences would require detailed engineering-level analysis, which is beyond the scope of this study.

An observed multicollinearity between circuits cannot be ignored in this analysis. Circuits with higher wildfire risk automatically have a higher chance of receiving investments. Therefore, a pre-existing risk and vulnerability of the circuit can mask a selection bias as well as lead to an overestimation of investment effectiveness or could portray correlation as a potential causation. The same principle can apply to weather variability as well, since many of these de-energization decisions are influenced by weather changes. To address potential weather-related variation, we implemented a proximity analysis designed to mitigate some of the limitations of the baseline cohort approach. By comparing circuits within similar geographic and environmental contexts, this method helps reduce the influence of localized weather shocks and other spatially correlated factors. Nonetheless, this adjustment does not fully eliminate the potential influence of confounding variables. A more comprehensive assessment would require additional studies explicitly designed to control for weather intensity, exposure, vegetation, topography, and other operational conditions through more detailed modeling.

## Supplementary Information

Below is the link to the electronic supplementary material.

Supplementary Material 1

## Data Availability

As identified in the links provided in the methods section, all geospatial and quantitative data used in this analysis are publicly available. Below is a list of the key sources, including their ownership, intended use case, and working links as of the time of publication (please see the references section for the wildfire mitigation plans and other key documents used to determine which circuits received investments).Although ICA (Integration Capacity Analysis) data is presented for all three IOUs, only two were used in the final analysis due to data restrictions. All three datasets are included here solely to help obscure which IOUs were used in the geospatial results. Information and links to data can be found in the data sources supplemental file. All data were processed using Python scripts, which can be provided by the corresponding author upon request.

## Abbreviations

CPUC: California Public Utilities Commission
EPSS: Enhanced powerline safety settings
HTFD: High fire threat district
IOUs: Investor-owned utilities
PG&E: Pacific Gas and Electric
PSPS: Public Safety Power Shutoffs
SCE: Southern California Edison
SDG&E: San Diego Gas and Electric
