# Supplementary material for: Evaluating investments in electricity resilience: evidence from California’s Public Safety Power Shutoff (PSPS) events
Source: Sci Rep. 2026 Sep 11;16:28465. doi: 10.1038/s41598-026-50735-4 (PMC13569834; doi:10.1038/s41598-026-50735-4)
Supplement: Supplementary file 1 [file 41598_2026_50735_MOESM1_ESM.docx]

| **Source name** | **Owner** | **Use Case** | **Link** |
| --- | --- | --- | --- |
| PSPS post event reports (and excel) | California Public Utilities Commission (CPUC) | Base data for use case (excel); reports to cross check reported outages in excel | https://www.cpuc.ca.gov/consumer-support/psps/utility-company-psps-reports-post-event-and-post-season |
| PSPS dashboard | CPUC | Cross-check data in excel from 2021 forward | https://capuc.maps.arcgis.com/  apps/dashboards/  ecd21b1c204f47da8b1fcc4c5c3b7d3a |
| Integrated Capacity Analysis Map | PG&E | To ensure a consistent format across the service area, circuit names reported during de-energizations were standardized by matching circuit names with report ICA circuit names (geospatial). De-energization events were then mapped to specific circuits.* | https://grip.pge.com/ |
| Integrated Capacity Analysis Map – Circuit Segments | SCE |  | https://drpep-sce2.opendata.arcgis.com/  datasets/SCE2::ica-layer?layer=2 |
| Integrated Capacity Analysis Map – Unavailable Circuit Segments | SCE |  | https://drpep-sce2.opendata.arcgis.com/ datasets/SCE2::ica-layer?layer=1 |
| Integrated Capacity Analysis Map | SDG&E |  | https://interconnectionmapsdge .extweb.sempra.com |
| California Electricity Utility Service Areas | California Energy Commission | Used to show the size of the three IOUs’ utility service areas in miles and as a proportion of all utility service areas across the state of California | https://cecgis-caenergy.opendata.arcgis.com/ datasets/CAEnergy::electric-load-serving-entities-iou-pou/about |
| High threat fire district map | CPUC | Used in coarse geospatial analysis to show | https://www.arcgis.com/home/item.html  ?id=986b9c5900b1424dac71b2f91b9b7475 |
